# The amount and value of work time of community medicine distributors in community case management of malaria among children under five years in the Ejisu-Juaben District of Ghana

**DOI:** 10.1186/1475-2875-11-277

**Published:** 2012-08-16

**Authors:** Peter Agyei-Baffour, Kristian S Hansen, Edmund N L Browne, Pascal Magnussen

**Affiliations:** 1 Department of Community Health, School of Medical Sciences, College of Health Sciences, Kwame Nkrumah University of Science and Technology, UPO, PMB, KNUST, Kumasi, Ghana; 2 Department of Global Health and Development, London School of Hygiene and Tropical Medicine, London, United Kingdom; 3 DBL-Centre for Health Research and Development, Faculty of Life Sciences, University of Copenhagen, Copenhagen, Denmark

**Keywords:** Community case management, Value of work time, Community medicine distributors, Malaria in children under five years, Ghana

## Abstract

**Background:**

The contribution of community medicine distributors (CMD) to prompt health service delivery in areas described as “hard-to-reach” is important but the value of their work time remains unknown and thus makes it difficult to design appropriate regular financial incentives to motivate them. This makes CMDs feel their efforts are not recognized. An attempt to estimate the value of 54 CMDs’ work time involved in community case management of malaria (CCMm) in a rural district in Ghana is presented.

**Methods:**

Time spent by CMDs on CCMm activities were recorded for a period of 12 months to determine the work-time value. Cost analysis was performed in Microsoft Excel with data from CMD records and at 2007 market price in Ghana.

**Results:**

A CMD spent 4.8 hours, [95% CI: 3.9; 5.3] on all CCMm-related activities per day. The time value of CMD work ranged from GH¢ 2.04 (US$ 2.24) to GH¢ 4.1 [US$ 4.6] per week and GH¢ 19.2 - 86.4 (US$ 21.10-94.95) per month. The gross wage outside CCMm as reported by CMD was GH¢ 58.4 [US$ 64.69] and value of foregone income of GH¢ 86.40 (US$94.95) per month, about 14-times higher than the monthly incentives of GH¢ 6.0 given by the CCMm programme.

**Conclusion:**

The value of work time and the foregone income of CMDs in CCMm are high and yet there are no regular and sustainable incentives provided for them. The results are significant to policy in designing incentives to motivate CMDs in large-scale implementation of CCMm.

## Background

The value of community medicine distributors’ (CMDs) effort in improving access to quality health care has long been recognized and their use encouraged [1-6]. The use of CMDs for community case management of malaria (CCMm) have been implemented in Uganda, Ghana and Nigeria, where volunteers were tasked to dispense pre-packed anti-malarials, organize behavioural change sessions and make referrals of severe fever cases. Under this intervention, caregivers accessing care pay a token to the CMD which is collected by the District Health Management Team to serve as revolving fund to replenish drugs [7-9]. It has been established that CMDs are effective as cultural brokers and health providers, leading to a reduction in inequity and improved health status in rural communities [10]. The benefits of using CMDs are both tangible and intangible [11]. But, assigning a “dollar-value” to a volunteer’s time remains debatable [12,13].

While it is difficult to collect relevant data for assigning a monetary value to a volunteer, there is a conflict in trying to monetarize a volunteer’s work without jeopardizing the voluntarism concept. It is argued that volunteers are not paid not because they are worthless but because they are priceless, and that volunteers help themselves by volunteering to help others [14]. The argument is that measuring a volunteer’s contribution in terms of money is just not right and may de-value the intrinsic reward of the work of a volunteer [15]. Judging from the meaning of the word “volunteer”, perhaps, the argument of de-monetarizing volunteer’s efforts appears to elicit the notion that voluntary activity is a “free good” with no accompanying costs [15].

However, voluntary organizations and some volunteers argue that volunteering is not cost-free, because recruiting, deploying, supporting and training volunteers requires time, money and human resources. They also contend that as we live in a world where human activities are rarely assessed and esteemed on the basis of their contributions to the well-being of others, it becomes imperative to attempt to measure the value of the effort of CMDs because we live in a context where only things we “value in dollars” have high priority for decision-makers [16,17].

It is important to note that the value of work time can provide a practical understanding of time input requirements and a quantifiable value to inform policy and to compare the cost effectiveness of groups of people involved in interventions. Information on the value of work time would impact on CMDs’ preparedness to work and stakeholders’ accountability of the resources entrusted, while at the same time providing justifications for garnering support for efficient utilization of CMDs for scaling up interventions. Many health programmes have not quantified the costs of involving CMDs or the value of their work time. It has been argued that having a regular incentive package for volunteers is not sustainable [17]. Apart from who should bear the cost and in what form should an incentive be, it is also important to answer pertinent questions such as “how much does it cost to engage a CMD per week per ‘X’ number of people per malaria (fever) case seen?” Or “how much will it cost the stakeholders to build health facilities and deploy trained staff to “hard-to-reach” areas compared with involving CMDs to provide service?” While the cost of the former is known in most cases, the cost of the latter remains unknown. Considering the role of CMDs in CCMm in Ghana and the fact that the National Malaria Control Programme is implementing a nationwide, home-based care of fevers, it is appropriate to measure the value of work time to guide the design of a sustainable and regular incentive package for the volunteers to facilitate smooth, large-scale implementation of CCMm interventions. This paper presents estimates of the amount of work time of CMDs spent on CCMm in a rural district of Ghana as well as valuations of the unpaid time offered by CMDs.

## Methods

### Study design

A longitudinal study was designed to estimate the number of hours of work time of CMDs in CCMm with artesunate-amodiaquine to manage presumptive malaria (fever) in children less than five years old in rural communities in the Juaben sub-district of Ejisu-Juaben district in Ghana. A total of 54 CMDs were selected and followed up for a period of 12 months (April 2006 to March 2007). The total population of the study area covered by the CMDs was 58,224 [18].

### Selection and deployment of distributors

The volunteers were selected by their communities based on trust and experience in community health activities. At least one CMD was selected in each community. After having an initial five-day training session, CMDs were introduced at community meetings in attendance with the research team. They were given their tool kits, which included medicine and stopwatches to time their involvement in CCMm activities.

### Medicines used in the studies

The Ghana national drug policy was strictly adhered to. Thus, the CMDs administered artesunate and amodiaquine (AS/AQ) in combination, at these dosage regimes: 25 mg/75 mg once daily for three days in children aged six to 11 months, and 50 mg/150 mg once daily for three days in children aged 12-59 months [19].

### Data collection

In-depth interviews were held periodically with CMDs. Time Tracking Sheets were used by CMDs to record the time they were involved in the various activities of CCMm [20]. The principal investigator and field supervisors supervised CMDs through spot checks and direct observations. CMDs did not incur any travel costs as caregivers presented their feverish children to them.

### Cost identification and estimate of the value of work time of distributors

For the estimation of cost incurred by CMDs, a total of 4,522 cases of presumptive malaria recorded by CMDs were included. The main activities considered were hours spent on receiving caregivers, drug administration, and recording of biodata of caregivers and their children who presented at CMDs’ houses, hours spent on follow-up visits and health education sessions. These were recorded on a special form. During direct observation, a list of tasks and a stopwatch were used to record the activity of the CMDs and to validate the time records in their books. The value of the time spent on an activity was estimated by multiplying the hours spent by the 2006 national minimum wage in Ghana, and a labourer wage in the study communities estimated during the study period [21,22].

### Measurement of time utilized by distributors

The amount of time used by CMDs was tracked on a form (Time Tracking Sheets) designed using Microsoft Excel. Each CMD was given a bound sheet and replaced as it was used up. CMDs were supplied with stopwatches. As a mother or caregiver presented her sick child, the stopwatch was set at zero and a reading taken after the CMD had finished the consultation. For quality assurance and validity assessment, a supervisor made spot checks and compared with CMDs’ recordings, which showed almost the same with correlation coefficient of 0.89. The time recorded by the CMDs were entered into Microsoft Excel and analysed.

The time spent on training was estimated as follows. CMDs were initially trained for five hours daily (9 am to 3 pm including one hour break) per day for five days) while supervisors were trained for three days for the same number of hours; and again for CMDs every month for the first three months and subsequently every two months.

Time spent in receiving medicines from supervisors were captured using the stop watches from the time the supervisor entered a CMD’s house until departure. The time taken for talking to the research team was excluded since this was part of the research and would not be part of routine service.

### Valuation of work time

The valuation of time spent by CMDs for their work in the CCMm programme was done according to a societal perspective: meaning that the opportunity costs to society of CMDs’ time was the relevant concept for this study. While CMDs are working and spending time in the CCMm programme, they are not able to perform their normal activities, and this is an opportunity cost to society. The level of opportunity costs of time depends on a range of factors, including what the normal activities are and the characteristics of the labour market. According to one approach, the time of volunteers or unpaid workers should be valued by a relevant market wage rate. This proposition is associated with an assumption of a perfectly competitive labour market where workers will be paid a wage rate equal to the value of their marginal product and that there will be no unemployment [23]. In another approach, a perfectly competitive market is not a realistic description of the labour market in most countries. If there is unemployment and labour surplus, the opportunity costs of time will tend to be lower than described above [24-26]. For instance, the work that CMDs would normally do if they were not allocating time for the CCMm programme could be done by unemployed or underemployed individuals.

For the present study, the time of CMDs was valued by different proxy measures following broadly the approaches described above.

First, all CMDs were assumed to work for eight hours per day, five days per week as labourers receiving the prevailing 2006 national minimum wage in Ghana. Based on this assumption, the 2006 national minimum wage of GH¢ 1.60 (US$ 1.76) per day, GH¢ 0.20 per hour, was used to calculate cost of the work done by CMDs. The gross value of CMDs’ effort was based on simple calculations of number of hours at work multiplied by minimum hourly wage per day and per week.

Another measurement technique involving the use of the average labourer wage, village wage rate, pertaining to the study communities at the time of the survey (estimated at GH¢3.50 per day or GH¢0.4 per hour, estimated as an average of GH¢45.0 for forest clearing and GH¢2.50 for less difficult farming activities) was used. Moreover, CMDs were asked how much extra (premium) they were prepared to pay to a labourer to carry out similar activities per day lost due to their involvement in CCMm activities, which they quoted as GH¢7.50 or GH¢0.90 per hour.

Finally, the value of work time was estimated in terms of foregone monthly income (based on reported income of CMDs), and reported mean monthly income of GH¢39.20, that is GH¢1.96, per day or GH¢0.30 per hour CMDs.

## Results

Table 1 presents the background characteristics of respondents involved in the study. Peasant farming was the main occupation. Crops grown were cash crops (cocoa, oil palm) and food (cereals, tubers and vegetables). About 70% of CMDs had basic education, 26% had secondary education. Most were married and were Christians. All had lived in the communities since birth and had been involved in community health activities as volunteers for 8.7 years on average.

**Table 1:** Background characteristics of distributors, n = 54

**Variable**	**Frequency (%)**
**Occupation**	
Farming	44 (81.4)
Other (petty trading, artisan, combined occupation)	10 (18.6)
***Education***	
Basic	38 (70.4)
Secondary	14 (25.9)
Post secondary	2 (3.7)
***Marital status***	
Married	48 (88.8)
Not married	6 (11.2)
**Sex**	
Male	46 (85.2)
Female	8 (14.8)
***Religion***	
Christian	48 (88.9)
Other	6 (11.2)
**Age**	
Mean	45.8
**Years in volunteering**	
Mean	8.7 (0.4)

### The work of the distributors

The job description of the CMD in CCMm was as follows (Table 2). On a typical day, the CMD checked his books, reviewed the opening balances and any outstanding entries, marked outstanding visits to caregivers previously visited, or the number of health education sessions to be held in the communities. When caregivers presented their children the CMD would record their background information. The children were examined and sponged with tepid water where necessary before any medication. Usually, the first dose was given by the CMD in the presence of the caregiver and she was instructed to do same the next and following days till all the medicine were given. The CMD counselled caregivers on the causes of fevers. The need for prompt action and the need for effective preventive measures, such as sleeping under insecticide-treated nets were emphasized. In the case of severe fever cases, CMDs prepared referral cards and assisted and, in some cases, accompanied caregivers to the nearest health facilities.

**Table 2:** Summary of activities of a CMD in a typical day in community case management of malaria

**Time of the Day**	**Activity**	**Remarks**
Early Morning	· Check records:	· Balance off previous activities
	· Number of health education sessions to organize	· Outstanding activities in the previous day are attended to
	· Attend to caregivers who present children	· Identify caregivers who need
	· Attend to personal activities (farm, trade, other work)	· home visit
	· May be recalled from work	· Whether to organize health educations and for whom?
	· Visit by research team	· Opening balance for the day
		· Records audited, educated and commended for good work, replenish stock
Evening	· Attend to caregivers who present their children	· Outstanding activities in the morning are continued
	· Home visits	· Records audited, educated and commended for good work, replenish stock
	· Organise health education	
	· Visit by research team	
Night	· Respond to emergencies	· Assist caregivers to nearest facility
	· Visit by research team	· Administer drugs
		· Offer counselling
		· Records audited, educated and commended for good work, replenish stock

An average of two caregivers visited a CMD per day. Some caregivers visited in the morning, in which case the CMD attended to them before leaving for his farm. It happened that he was sometimes called from his farm to attend to a child with fever. Caregivers either sent for the CMD from the farm, market place, school (CMDs who are teachers) or went to these places themselves with their children. This interrupted the economic activities and income of the CMD. In the case of organizing heath education sessions, the CMD started the preparations by identifying his target audience and assembling materials needed. If the health education was to be held for the whole community, he discussed it with the chief and elders who then assisted in publishing through the *gong-gong* beating or town criers. When he returned from the farm, school or market, the CMD continued with his household chores and stopped to attend to caregivers bring febrile children. Follow-up visits by the CMD were generally made in the evening. Caregivers also visited the CMDs at night, so in reality CMDs provided a 24 hour service.

### The value of work time spent by distributors on community case management of malaria

Table 3 shows the various activities and time spent by CMDs on CCMm. Data shown are mean values based on records for 4,522 cases of fever. The number of children seen by each CMD per day was 1.8 and on average 24 min was spent on each case making a total of 43 min/day. CMDs on average did 2.5 behavioural change sessions per day, spending 12.1 min per session to 30 min/day. Follow-up visits carried out were 2.6/day and this activity equalled 102 min/day). In total a CMD spent 4.8 hr/day on CCMm-related activities.

**Table 3:** Summary of activities and time spent by CMD per day

**Variable**	**Mean**	**95% CI**
Children seen per day	1.8	1.8-1.9
Time spent on seeing children (hrs)/day	0.4	20.9-21.1
No of Health education activities/day	2.5	2.3-2.6
Time spent on IEC (hrs)	0.2	11.4-12.8
Follow-ups/day	**2.6	2.4-2.8
Hours on follow-up/child	1.7	1.5-1.8
Total hours spent on CCMm activities/day	*4.8	3.9-5.3

* excludes hours spent during night visits by caregivers. Computed from 1936 observations from CMD time tracking sheets, hours could not be validated; ** a child was likely to be followed up more than once.

### Measurement and valuation of the value of work time

Table 4 presents the estimated value of work time of CMDs in CCMm expressed in 2006 market price. The value of work time was valued using GH¢0.2 (minimum wage rate) per hour and GH¢0.4 (the estimated village labour wage (VLW) rate per hour) for eight working hours per day.

**Table 4:** Estimated opportunity cost of CMDs’ time

**Time/Cost**	**Mean total time (minutes)**		**Weekly Value of work time by valuation method**			
**Time cost**			National minimum wage*	Village labourer wage**	Reported monthly Income***	how much extra (premium) ****
Minutes/child	1.8x24	43	0.7	1.22	0.88	3.15
Minutes/BCC session	2.5x12.1	30	0.5	0.86	0.63	2.25
Minutes on follow up/child	2.6x1.7	102	1.7	2.98	1.53	7.65
^**a**^**Monthly opportunity cost of time**	**20 working hours -**		**4.8 (5.3)**	**9.6(10.67)**	**14.64 (16.27)**	**21.60 (23.74)**

^a^Estimated based on 4.8 working hours/day and 5 working days per week*; As per the 2007 Ghana national minimum wage of (1.6/8 hours = 0.20]; ** As per an estimated village labourer wage of (3.5/8 hours = 0.4). *** As per reported income, 1.96/8 = 0.25 **** As **per** how much extra (premium) they would pay due to child’s sickness7.5/8 = 0.91; US Dollars in parenthesis. **(1 US$ = GH**¢ 0.**90).**

From Tables 3 and 4, the value of CMDs’ work for 4.8 hours per day multiplied by five days equals 24 hours per week, 24 hours x GH¢0.20 per hour based on minimum wage of GH¢1.60, amounts to GH¢4.80 (US$ 5.33) per week. This amounts to GH¢19.20 (US$21.30) per month. On the other hand, on the basis of the VLW, the value of a CMD’s work for 4.8 hours per day multiplied by five days equals 24 hours per week; 24 hours x GH¢0.40 per hour based on VLW rate of GH¢3.50 amounts to GH¢9.60 (US$10.67), GH¢38.40 (US$42.70) per month on an exchange rate of (US$1 = GH¢0.90). In the case of reported income, using the same assumptions, the value of a CMD’s effort is even higher; 24 hours x 0.61 amounting to GH¢14.64 (US$16) per week, GH¢58.40 (US$64.90) per month and that of the foregone value is 4.8 hours x 5 days x GH¢0.90 amounting to GH¢21.60 (US$23.74) per week or GH¢86.40 (USD$94.95) per month.

## Discussion

A follow-up of 54 CMDs was performed in CCMm for a period of 12 months using time and motion study sheets [19] to document time spent on key activities of CCMm to estimate the value of work time of CMDs in CCMm. Volunteer CMDs work for 4.8 hours, about 60% of a standard work day of eight hours, yet the economic value of their effort has rarely been explored [12,13]. The question is how much monetary reward in Ghana Cedis do CMDs earn from CCMm activities? Accurate estimates of the value of volunteer work may be useful to decide on appropriate incentives packages for CMDs.

CMDs had on average nine years’ experiences as volunteers. A majority had only basic education, which means that regular training should be part of an incentive package. All CMDs were adult, married and had children, which meant that economic pressure as bread winners may be a disincentive to volunteering in CCMm.

Although CMDs did not receive regular remuneration for their CCMm work, they enjoyed social capital [1,11] which was not available in the formal sector. Direct benefits to volunteers were identified as self esteem (“village doctors”), increased skills, recognition, and packed lunch during training. They also benefitted from non-salary remuneration such as bicycles, torch lights, boots and raincoats and recognition at health facilities [7].

The CMDs’ economic activities were interrupted by CCMm activities. Compared with the incentive given in CCMm, the value of “normal” work time was four times higher. This was likely to affect their income flow and adversely affect the upkeep of their homes. In CCMm, CMDs were paid 6.4 times lower than their reported monthly income. The use of minimum wage underestimated the value of the work time of CMDs by GH¢1.9. This corroborates with similar findings by Asenso-Okyere and Dzator [22], when they estimated the household cost of seeking care in two districts in Ashanti region of Ghana, suggesting that the use of village wage rate may be preferred to estimating the time value of workers in health programmes.

There were limitations in the study. The study used reported monthly income of CMDs. This was difficult to validate as CMDs had no income records. Judging from the fact that CMDs had lower education level, the possibilities of errors in their records from which the value of time was estimated could not be ruled out. Also, social desirability bias may have influenced CMDs’ responses to the survey questions. The value of work time was estimated in terms of foregone monthly income (based on reported income of CMDs). This was difficult to validate in a subsistence economy where there is virtually no record on income. The study did not include the cost of community meetings as these were not available under the CCMm. It excluded work hours at weekends as rate of part-time work was not available. It is important to caution that in African society, especially Ghana, one is obliged to greet whoever he meets and this can sometimes lead to more than 10 minutes’ conversation. It is possible the CMD wrongly included these in time utilized.

The average follow-up was higher than the average number of children seen. This was possible as some children were visited more than once. These factors limit the generalizability of the results to some extent. Notwithstanding, the study makes a case for the institutionalization of the efforts of CMDs especially in areas described as “hard-to-reach” as means of recognizing their effort in health care delivery.

## Conclusions

In conclusion, CMDs represents a cheap resource in otherwise resource-constrained settings to assist in improving prompt access to established health interventions such as CCMm. The study concludes that if we cannot establish standard ways of compensating these valued workers, then we face the real possibility of losing their contributions in CCMm and in rural areas where their services are needed most. Future studies should provide a means of validating income records of CMDs, and a costing template to capture all cost elements, both financial and non-financial, and death averted, and disability life averted in CCMm.

## Competing interests

The authors declare that they have no competing interests.

## Authors’ contributions

PM co-ordinated, monitored and supervised the study, participated in data analysis and critically reviewed the manuscript. KSH supervised and participated in the design, statistical analysis and preparation of the manuscript. ENLB contributed to the design, supervised field activities and participated in the preparation of the manuscript. PA-B conceived and led the study, participated in its design, implementation, statistical analysis, reporting and preparation of the manuscript. All authors read and approved the final manuscript.
